# Something Old, Something New, Something Borrowed; How the Thermoacidophilic Archaeon *Sulfolobus solfataricus* Responds to Oxidative Stress

**DOI:** 10.1371/journal.pone.0006964

**Published:** 2009-09-16

**Authors:** Walid S. Maaty, Blake Wiedenheft, Pavel Tarlykov, Nathan Schaff, Joshua Heinemann, Jim Robison-Cox, Jacob Valenzuela, Amanda Dougherty, Paul Blum, C. Martin Lawrence, Trevor Douglas, Mark J. Young, Brian Bothner

**Affiliations:** 1 Department of Chemistry and Biochemistry, Montana State University, Bozeman, Montana, United States of America; 2 Thermal Biology Institute, Montana State University, Bozeman, Montana, United States of America; 3 Department of Microbiology, Montana State University, Bozeman, Montana, United States of America; 4 Department of Plant Sciences, Montana State University, Bozeman, Montana, United States of America; 5 Department of Mathematical Sciences, Montana State University, Bozeman, Montana, United States of America; 6 George Beadle Center for Genetics, University of Nebraska, Lincoln, Nebraska, United States of America; Yale University, United States of America

## Abstract

To avoid molecular damage of biomolecules due to oxidation, all cells have evolved constitutive and responsive systems to mitigate and repair chemical modifications. Archaea have adapted to some of the most extreme environments known to support life, including highly oxidizing conditions. However, in comparison to bacteria and eukaryotes, relatively little is known about the biology and biochemistry of archaea in response to changing conditions and repair of oxidative damage. In this study transcriptome, proteome, and chemical reactivity analyses of hydrogen peroxide (H_2_O_2_) induced oxidative stress in *Sulfolobus solfataricus* (P2) were conducted. Microarray analysis of mRNA expression showed that 102 transcripts were regulated by at least 1.5 fold, 30 minutes after exposure to 30 µM H_2_O_2_. Parallel proteomic analyses using two-dimensional differential gel electrophoresis (2D-DIGE), monitored more than 800 proteins 30 and 105 minutes after exposure and found that 18 had significant changes in abundance. A recently characterized ferritin-like antioxidant protein, DPSL, was the most highly regulated species of mRNA and protein, in addition to being post-translationally modified. As expected, a number of antioxidant related mRNAs and proteins were differentially regulated. Three of these, DPSL, superoxide dismutase, and peroxiredoxin were shown to interact and likely form a novel supramolecular complex for mitigating oxidative damage. A scheme for the ability of this complex to perform multi-step reactions is presented. Despite the central role played by DPSL, cells maintained a lower level of protection after disruption of the *dpsl* gene, indicating a level of redundancy in the oxidative stress pathways of *S. solfataricus*. This work provides the first “omics” scale assessment of the oxidative stress response for an archeal organism and together with a network analysis using data from previous studies on bacteria and eukaryotes reveals evolutionarily conserved pathways where complex and overlapping defense mechanisms protect against oxygen toxicity.

## Introduction

Oxidative stress is a universal phenomenon experienced by both aerobic and anaerobic organisms from all three domains of life [1] and is primarily the result of excess reactive oxygen species (ROS) in the cell. Reactive oxygen species (ROS) are generated in a number of ways, including incomplete oxygen reduction during respiration or exposure to environmental factors such as light, increased partial pressure of oxygen, and metals. ROS such as super oxide (O_2_^.−^), hydroxyl radical (OH^.^) and hydrogen peroxide (H_2_O_2_) chemically damage DNA, RNA, protein, lipids, and cofactors [2]–[4]. Oxidative stress is of general interest because of the link between chemical assault on biomolecules and diseases [5], [6], organization of microbial communities, the environmental fate of chemicals, and the evolution of oxygenic life on earth.

Cellular defense mechanisms to counteract oxidation include enzymes and antioxidant molecules (e.g. superoxide dismutases, catalases, peroxidases, thioredoxins, peroxiredoxins and glutathione) [7]–[10]. The interplay between these and other cellular components is complex, therefore, it has been suggested that a systems biology approach is critical to understanding how the system is orchestrated [11]–[14]. For example, superoxide dismutases convert superoxide anions to hydrogen peroxide [15], which is in turn reduced by catalases, peroxiredoxin and peroxidases as part of a multi-step branching pathway [16], [17]. Numerous studies have investigated individual enzymes and their pathways in the detoxification of ROS within eukaryotes and bacteria. Relatively few studies have investigated oxidative stress response in Archaea and an overall comparison between the three domains of life is lacking.

Cellular response to H_2_O_2_ has general similarities and specific differences across domains. Peroxide-inducible genes in *E. coli* such as DPS (DNA binding protein in nutrient starved cells [18]) and catalase are controlled by the regulator OxyR. A second set of OxyR-independent genes, respond to general ROS, revealing that multiple pathways respond to oxidative stress [19]. In the anaerobic Gram-negative symbiont *Bacteroides fragilis*, oxidative stress induces the expression of peroxidases, catalase, DPS, ferritin, superoxide dismutase, and bacterioferritin [20]–[22]. The Gram-positive soil bacterium *Bacillus subtilis* uses a different set of defense mechanisms composed of scavenging enzymes as well as protection and repair systems from the PerR and the Fur regulon [23]. In the yeast *Candida albicans*, peroxide stress induced the expression of 21 proteins with known antioxidant functions including catalase, thioredoxin reductase, oxidoreductases, and 12 heat shock proteins under the regulation of Cap1p [24]. These response mechanisms can be sophisticated as in the case of the fission yeast *Schizosaccharomyces pombe*, which has two pathways, Pap1 and Sty1, which are triggered by different H_2_O_2_ concentrations [25].

Archaeal organisms also possess multiple oxidative stress response pathways, although this is based on limited data from studies that for the most part were focused on single proteins or pathways. One mechanism involves nonheme iron proteins such as rubrerythrin, which has been shown to have antioxidant properties in the hydrogenotrophic methanogen euryarchaeotes *Methanothermobacter thermautotrophicus* [26], the anaerobic sulfate-reducing bacterium *Desulfovibrio vulgaris,* and the bacterial pathogen *Porphyromonas gingivalis* [27], [28]. Rubrerythrin in *D. vulgaris* functions as a terminal component of NADH peroxidase in the reduction of hydrogen peroxide to water [27], [29]. The protein is a homodimer that contains both a rubredoxin-like [Fe(SCys)_4_] center and a non-sulfur, oxo-bridged di-iron site [30]. A second anti-oxidative damage pathway in Archaea involves DPS-Like protein (DPSL). DPSL proteins are a phylogenetically distinct subclass of di-iron carboxylate proteins that assemble into a homo-dodecameric cage ∼10 nm in diameter and are widely distributed in phylogenetically diverse prokaryotes [9], [31]. The protein structures are homologous to the multimeric assemblies formed by the iron-mineralizing family of ferritin proteins [9], [31] and DPSL from *Pyrococcus furiosus* and *S. solfataricus* have been biochemically characterized [9], [31], [32]. This ferritin-like protein uses H_2_O_2_ as an oxidant instead of O_2_, effectively eliminating both hydrogen peroxide and ferrous iron that can contribute to the generation of hydroxyl radicals via the Fenton reaction [33], [34]. It has also been shown that the *S. solfataricus dpsl* gene is up-regulated in response to H_2_O_2_ and iron depletion.

A driving force for this work is an interest in evolutionarily conserved mechanisms for managing oxidative stress. Hyperthermophilic archaea are deeply rooted in the rDNA gene based tree of life and as such may harbor ancient mechanisms that shed light on the origin and evolution of the oxidative stress response in contemporary life. *S. solfataricus* is a thermoacidophilic Crenarchaeota that grows optimally at ∼pH 3.0 and at temperatures ranging from 72–85°C. The complete genome sequence for the P2 strain of *S. solfataricus* is available and the development of both genetic [35], [36] and biochemical [37] tools have contributed to the development of *S. solfataricus* as a model organism for examining the archaeal lifestyle and life in high temperature environments. In this study, we have combined transcriptome, proteome, gene disruption, protein interaction, and chemical activity to establish the oxidative stress network in *S. solfataricus*. This work has allowed a system-wide network to be constructed. At the center of this network is a novel protein complex that contains multiple proteins that could function in concert to remove ROS. The archaeal oxidative stress network described here was combined with previous work on oxidative stress in bacteria and eukaryotes to construct a protein family network representing all three domains of life (Archaea, Bacteria and Eukarya).

## Materials and Methods

### Culturing of *S. solfataricus*

Liquid cultures of *S. solfataricus* (P2), were grown aerobically in DSMZ media 182 (22.78 mM KH_2_PO_4_, 18.90 mM (NH_4_)_2_ SO_4_, 0.81 mM MgSO_4_, 1.7 mM CaCl_2_, 0.2% Yeast Extract) pH adjusted to 2.8 with 6N H_2_SO_4_. All cultures were grown in long neck Erlenmeyer flasks at 80°C. Hydrogen peroxide was administered to stress cultures, to a final concentration of 30 µM. Hydrogen peroxide concentrations were determined using the molar extinction coefficient (43.6 M^−1^ cm^−1^) at 240 nm [38].

Three liters of DSMZ media 182 (pH∼2.8) was inoculated with 15 mls of a late-log phase (OD_650_ 0.52) *S. solfataricus* culture and then divided evenly between three, 2-liter long neck culturing flasks. At 62.5 hrs after the start of culturing (OD_650_ ∼0.3), 20 mls of each 1 liter culture was removed and placed in a separate 50 ml flask as non-H_2_O_2_ stressed growth controls. An additional 50 ml aliquot was collected from each culture and used for protein and RNA isolation (t = 0). The three separate cultures (∼930 mls) were then treated with hydrogen peroxide (final concentration of 30 µM). At 1, 2, 4, 8, 15, 30, 45, 60, 75, 90, 105, 120, 150 and 195 mins post H_2_O_2_ addition, 50 ml aliquots were removed for protein and RNA isolation.

### RNA Isolation and Northern blot analysis

Total cellular RNA was extracted from *S. solfataricus* cells, according to the Qiagen's RNeasy midi protocol, with an on-column DNase step (Valencia, CA). Total RNA concentrations were estimated using a Nanodrop spectrophotometer (OD_260/280_). RNA quantity and quality was independently assessed by visualization on a 1.5% agarose (wt/vol) formaldehyde gels and on an Agilent 2100 Bioanalyzer (Agilent Technologies, Palo Alto, CA). ∼1.2 µg of total RNA was separated by electrophoresis in a 1.5% agarose (wt/vol) formaldehyde gel and transferred to GeneScreen membranes as recommended by the manufacturer (NEN, Wellesley, MA). RNA was membrane-crosslinked in a UV Stratalinker (Stratagene, La Jolla, CA). Blots were probed with ^32^P-labeled *Ssdpsl* PCR products (Ready-To-Go Labeling Kit, GE Healthcare).

### Microarray analysis

The NimbleGen *S. solfataricus* (P2) microarray platform was used to assess the organism's transcriptional response to 30 µM H_2_O_2_. Unless noted, all experiments were repeated three times. The NimbleGen oligo expression array includes 15 (24mer) probes per target, each of which is duplicated in two separate blocks and represents 2,977 *S. solfataricus* genes (Madison, WI). RNA was isolated from exponential phase *S. solfataricus* cultures 30 minutes after addition of H_2_O_2_. The RNA from each sample was biotinylated and hybridized to separate chips. Signal intensity of each feature (ORF) was evaluated using a streptavidin conjugated Cye3 stain. Each RNA sample was used as the template for the incorporation of a derivatitized nucleotide (amino allyl dTTP) in a cDNA synthesis reaction that included; 1.5 µg of total RNA, 5 µg random hexamer primers, 5×RT buffer, 0.5 mM dNTP mix (4∶1 amino allyl dUTP/dTTP) and Superscript II and dithiolthreitol (DTT). All products except aadNTPs (Sigma, St. Louis, MO) and dNTPs (Promega, Madison, WI) were purchased from Invitrogen (San Diego, CA). Primers and RNA template were incubated for 10 min. at 70°C, cooled on ice for 2 min. prior to primer extension and allowed to react at 42°C for 2 hrs. RNA template was subsequently degraded in 20 mM EDTA and 50 mM NaOH. cDNAs larger then ∼70 nt were purified using MinElute (Enzymatic Clean up Kit) filtration columns (Qiagen). cDNA libraries were fluorescently labeled at room temperature, in the dark, with either Cy3 or Cy5 (Amersham), for 1.5 hr. Excess dye was removed using the MiniElute kit. Slides were prehybridized in 50 mls of 5xSSC, 0.1% SDS, 0.5 g BSA for 40 mins. at 42°C. Labeled cDNAs were combined in a hybridization mixture (27 µl formamide, 15 µl 20xSSC and 0.6 µl 10% SDS), applied to the array, covered with a lifter slip (Erie Scientific, NH) in a hybridization chamber (Arrayit, CA) and incubated at 42°C overnight (16–20 hrs). Untreated-Cy5 and H_2_O_2_ treated-Cy3 sample pairs were hybridized to a single glass slide. A second slide was hybridized with the reverse dye-sample pairing (untreated-Cy3 and H_2_O_2_ treated-Cy5), to account for any fluorescent dye biases. This approach was repeated with RNA from the second culture increasing the representation of each probe in each condition to eight. After hybridization, each microarray was washed with 2xSSC, 0.1%SDS for 5 minutes at 42°C, followed by 0.1xSSC, 0.1%SDS for 20 minutes at 42°C and rinsed in 5 times in 0.1xSSC at room temperature. After hybridization, arrays were scanned at 10 µm resolution using an Agilent scanner, Model G2565B (Agilent Technologies). Primary data collection and analysis were carried out using GenePix Pro 6.0 (Axon Instruments).

GenePix Pro 6.0 was used to align the grid and evaluate spot quality (Molecular Devices). The criteria for flagging were a signal-to-noise ratio of <3 in both of the channels or a regression ratio of <0.2 times or >1.8 times the ratio of the medians. These criteria were designed to remove features that were similar in intensity to the background or were not uniform. Data from the NimbleGen array analyzed separately using GeneSpring software (Silicon Genetics, Redwood City, CA) and represents 3 biological replicates with two technical replicates each (total n = 6). First, the data was filtered for baseline average raw signal intensity of at least 200, followed by filtering for a minimal fold change difference of 1.5. Data was normalized per chip to 50th percentile and per gene to median. Subsequent ANOVA testing was performed using a student's t-test and Benjamini & Hochberg multiple test correction with a false discovery rate of 5% [39]. All data is MIAME compliant and that the raw data has been deposited in a MIAME compliant database. The entire NimbleGen array data set has been deposited in the Gene Expression Omnibus database (GEO) at NCBI under accession number GPL7538 (http://www.ncbi.nlm.nih.gov/projects/geo/query/acc.cgi?acc=GPL7538).

### Construction and characterization of the *S. solfataricus dpsl* mutant

A *dpsl* loss of function mutation (*-ssdpslko/lacS*) generated by *lacS* insertion was constructed in *S. solfataricus* strain PBL2025 using linear recombination [35], [36], [40]. To simplify the process, a new strategy was employed requiring three simultaneous crossovers between two PCR products and the homologous region of the chromosome. The PCR products were produced by overlap extension PCR fusing either the 5′ or 3′ end of *dpsl* and its flanking sequences together with the *lacS* gene (SSO3019) resulting in fragments of about 1.5 kb. The *lacS* insert was placed 50 nt into the *dpsl* open reading frame. The two PCR products were then co-transformed into electrocompetent cells as described [35] and homologous recombinants recovered by enrichment in a minimal lactose medium as described [40]. Clonal recombinant cultures were established by colony purification on a solid complex medium containing tryptone (0.2% w/v). The *dpsl* allele was examined in three purified isolates by PCR using primers complementary to regions located 5′ and 3′ to the *dpsl* coding region. The uninterrupted allele produced an amplicon of 1 kb while the *lacS* disrupted allele produced an amplicon of 2.8 kb.

*S. solfataricus* 98/2 and insertion disruption mutant (-*ssdpslko/lacS*) strains were cultivated in a liquid complex medium (tryptone 0.2% w/v) at pH 3.0 and 80°C in screw capped flasks with agitation. Cultures were treated during exponential growth with H_2_O_2_ at final concentrations of 20 µM, 25 µM, and 30 µM at a cell density of 10^8^/ml. After 2.5 doublings, samples from treated and untreated cultures were harvested at cell densities of 5×10^8^/ml and processed for Dpsl analysis. Prior to electrophoresis, samples were adjusted to 2% (w/v) SDS and 3 mM β-mercaptoethanol and boiled for 10 minutes. Proteins were resolved in 100 µg amounts by SDS-PAGE with 14% (w/v) resolving gels and 5% (w/v) stacking gels and PageRuler prestained molecular mass standards (Fermentas). Chemiluminescent western blot analysis was performed using the ECL system (GE Healthcare) as previously described [41].

### Protein preparation

The cells were harvested by centrifugation at 5000×g at 4°C for 15 minutes and washed with ice-cold PBS (pH 7.4). Cells were broken by freeze and thaw followed by sonication in urea lysis buffer (30 mM Tris-HCl pH 8.5, 7 M urea, 2 M thiourea, 4% CHAPS, 1% ASB-14, 50 mM DTT, 0.5% IPG carrier ampholytes and protease inhibitor cocktail). After the supernatant was clarified by centrifugation, soluble proteins were purified and concentrated by precipitation with 5 volumes ice-cold acetone, and resolubilized for 1 h in urea lysis buffer. Protein concentration was measured with the RC/DC Protein Assay Kit (Bio-Rad). Samples were kept frozen until use.

### 2D-DIGE analysis

Soluble *S. solfataricus* protein fractions were labeled with CyDyes according to the manufacturer's protocol. Briefly, 50 µg of each protein extract was labeled separately at 0°C in the dark for 30 min with 400 pmoles of the N-hydroxysuccinimide esters of cyanine dyes (Cy3 and Cy5 CyDyes; GE Healthcare). The internal standard, an equimolecular mixture of all the protein extracts, was labeled with Cy2. Total protein labeled with Cy2, Cy3 and Cy5 for matched control and H_2_O_2_ stressed samples were combined and mixed with the urea lysis buffer. 2-DE was performed as described elsewhere [42], [43], using precasted IPG strips (pH 3–11 NL, non-linear, 24 cm length; GE Healthcare) for the first dimension (IEF). Typically, 150 µg of protein (50 for each dye) was loaded on each IPG strip and IEF was carried out with the IPGPhor II (GE Healthcare). Focusing was carried out at 20°C, with a maximum of 50 µA/strip. Active rehydration was achieved by applying 50 V for 12 h. This was followed by a stepwise progression of 500 V up to 8000 V for a total of 44,000 Vhr. After IEF separation, the strips were equilibrated twice for 15 min with 50 mM Tris-HCl, pH 8.8, 6 M Urea, 30% glycerol, 2% SDS and a trace of bromophenol blue. The first equilibration solution contained 65 mM DTT, and 53 mM iodoacetamide was added in the second equilibration step instead of DTT. Second-dimension SDS-PAGE was performed in Dalt II (GE Healthcare) using 1 mm-thick, 24-cm, 13% polyacrylamide gels, and electrophoresis was carried out at a constant current (15 mA/gel for ∼16 h at 20°C). Electrophoresis was completed once the bromophenol blue dye front reached the bottom of the gel. ProQ™ Diamond Phosphoprotein Gel Stain (Invitrogen), SYPRO^®^ Ruby (Bio-Rad), and Coomassie^®^ Brilliant Blue (Thermo Scientific) stains were used according to the manufacturer's instructions. Cydye swapping was not performed because the regulated spots in this study were not among the ones that have abnormal labeling behavior (Maaty et al., unpublished data).

### Image acquisition and analysis

After electrophoresis, gels were scanned using the Typhoon Trio Imager according to the manufacturer's protocol (GE Healthcare). Scans were acquired at 100 µm resolution. Images were subjected to automated difference in gel analysis using Progenesis SameSpots software version 3.1 (Nonlinear Dynamics Ltd.). The Cy3 gel images were scanned at an excitation wavelength of 532 nm with an emission wavelength of 580 nm, Cy5 gel images were scanned at an excitation wavelength of 633 with an emission wavelength of 670 nm, while the Cy2 gel images were scanned at an excitation wavelength of 488 nm with an emission wavelength of 520 nm. Gel spots were co-detected as DIGE image pairs, which were linked to the corresponding in-gel Cy2 standard. After scanning, the gels were stored in 1% acetic acid at 4°C until spot excision. Spots were identified and volumes were quantified using Progenesis SameSpots software. Raw volumes were exported from Progenesis and read into R (R Development Core team). Cy3 and Cy5 volumes were standardized to Cy2, then t-tests were performed for each spot using Log_2_(Cy3/Cy5) normalized to the median on each gel. [Note: This is equivalent to a matched-pairs t-test and does not assume that Cy3 and Cy5 volumes are independent on a given spot]. Q-values were computed using the q-value package [44], [45] and fdr was computed with the Benjamani and Hochberg correction [39].

### Protein identification

Protein spots of interest were excised from the gels, washed, in-gel reduced and S-alkylated, followed by digestion with porcine trypsin (Promega) overnight at 37°C [43], [46]. The solution containing peptides released during in-gel digestion were transferred to sample analysis tube prior to mass analysis. LC/MS/MS used an Agilent XCT-Ultra 6330 ion trap mass spectrometer fitted with an Agilent 1100 CapLC and ChipCube (Agilent Technologies). Samples were trapped and desalted on the Zorbax 300SB-C18 Agilent HPLC-Chip enrichment column (40 nl volume) in 5% acetonitrile 0.1% formic acid delivered by an auxiliary CapLC pump at 4 µl/min. The peptides were then reverse eluted and loaded onto the analytical capillary column (43 mm×75 µm ID, also packed with 5 µm Zorbax 300SB-C18 particles) connected in-line to the mass spectrometer with a flow of 600 nl/min. Peptides were eluted with a 5 to 90% acetonitrile gradient over 16 min. Data-dependent acquisition of collision induced dissociation tandem mass spectrometry (MS/MS) was utilized. Parent ion scans were run over the *m/z* range of 200 to 2,200 at 24,300 *m/z*-s. MGF compound list files were used to query an in-house database using with MS and MS/MS ion mass tolerances of 1.2 and 0.5 amu respectively. Positive identification required two significant peptides based on MASCOT (Matrix science, London, UK) MOWSE scores greater than 32 (p<0.05), however, most protein scores were significantly above the minimum criteria. Protein fold recognition using 1D and 3D sequence profiles coupled with secondary structure and solvation potential were performed using PHYRE (Protein Homology/analogY Recognition Engine; http://www.sbg.bio.ic.ac.uk/phyre/index.cgi).

### Protein thiol reactivity

*S. solfataricus* cells H_2_O_2_-treated for 0, 30 or 105 min were resuspended in PBS (pH 7.4). Cells were lysed by sonication (three, 30 sec cycles at 50% duty cycle with a power output of 2; Branson Sonifier); the protein supernatants were clarified by centrifugation at 20,000×*g* for 12 min at 4°C and collected. The cell lysates were labeled with the fluorescent sulfhydryl-modifying reagent 4,4-difluoro-3,5-bis(4-methoxyphenyl)-8-(4-maleimidylphenyl)-4-bora-3a,4a-diaza-*s*-indacene (BODIPY^®^ 577/618 maleimide; Invitrogen). The reagent concentration was adjusted to 1 mg/ml and the reaction was carried out for 2 hours in the dark in the presence of 1% SDS. Samples were mixed with 4×SDS sample buffer (non-reducing conditions) before separation over mini 4–20% gradient SDS-PAGE gel. Labeled mixtures were run on SDS gel in triplicate. Fronts were run off the gels to remove unreacted dye and obtain cleaner fluorescence images. Fluorescence image was obtained on Typhoon Trio Imager (GE Healthcare) using green laser (532 nm) with 610 nm filter at 400 V. Then the gel was stained with Coomassie^®^ Brilliant Blue and scanned in 48 bit color mode at 600 dpi resolution without color correction. Scans were stored as TIF images with no compression. Background subtraction was done using ImageJ (National Institutes of Health; version 1.39 m). Lanes from triplicate experiment were averaged using Image Calculator and Region of Interest (ROI) manager. Graph of fluorescent intensity versus pixel width was plotted using Microsoft Excel 2003 (Microsoft) software.

### Isolation and identification of SsDPSL complex

Size-exclusion chromatography was performed over a Superose-6 column at a flow rate of 0.5 ml per min. Total protein was monitored at 280 nm and fractions were collected for western blot analysis. DPSL expressed and purified from *E. coli* [9] was used as a control for size exclusion chromatography and as a protein source for immobilization on agarose beads. SsDPSL immobilization on the AminoLink Plus Coupling Gel (4% cross-linked beaded agarose, 50% slurry) was performed according to the manufacturer's instructions (Pierce; Rockford, IL). The DPSL protein was coupled using the pH 10 coupling procedures and using AminoLink Reductant Cyanoborohydride solution (NaCNBH_3_). After coupling, the DPSL-agarose beads were mixed with total *S. solfataricus* total cell lysate in presence of protease inhibitors for 2 hrs at 4°C. The beads were washed three times with 50 mM MES pH 7.2, 100 mM NaCl, 0.3% NP-40, and 2 mM EDTA to remove nonspecific proteins. At this point, proteins were either eluted using pH 2.5 glycine or digested with trypsin without elution after adjusting the pH to 8.5. Specifically bound proteins were eluted using 0.2 M glycine pH 2.6. A mock column of agarose beads only served as a negative control for nonspecific binding. Samples from before and after elution were subjected to trypsin digestion and mass spectrometry analysis as described above.

### Network Analysis

In addition to global profiling of the oxidative response in *S. solfataricus* a comparative analysis across all three domains of life was made using the data presented here and that from previously published transcriptomics and proteomics experiments. Comparable data for Eukarya [13], Bacteria [19], [23], and Archaea (our own) were compiled for this study. Data sets were converted into a congruent format by replacing official gene symbols, locus tags, or GI accession numbers, with Entrez Gene IDs. The DAVID (Database for Annotation, Visualization and Integrated Discovery) gene ID conversion tool was used, along with the ID converter tool g:Convert from the bioinformatics tool g:Profiler [47]. Once the gene list was in a common format, we used the DAVID functional annotation tool to search for their COGs, Pfams, and Gene Ontologies in order to help develop an interaction network. In order to help visualize the interaction network of the oxidative stress across domains we employed Cytoscape 2.6.1. [48], [49].

Protein family categorization provided 83 percent coverage of the Entrez Gene IDs submitted to DAVID's functional annotation tool. While some of the other types of annotation provided better coverage of the 858 genes submitted to DAVID, Pfam annotation allowed for assignment of broader definitions per each gene. The Pfams generated by DAVID were also more specific than necessary and were manually given broader names based on Wellcome Trust Sanger Institute's (WTSI) online description of each Pfam. In addition clans, or groups of homologous Pfams, were included in the oxidative stress comparison of all three domains [50]. To avoid producing extensive interactions, clans which contain large groups of Pfams were not assigned to their respective genes. The network map elucidating the interaction between the regulation of genes in all three domains and the specific type of regulation was generated using Cytoscape's Spring Embedded layout.

## Results and Discussion

**Figure 1:**
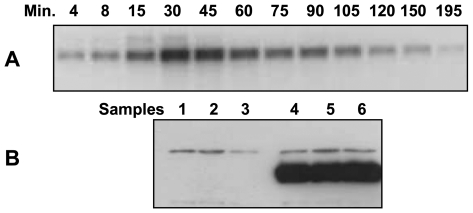
Treatment of *S. solfataricus* with 30 µM H_2_O_2_ leads to an up-regulation in transcription and translation of DPSL. A) Time course northern blot analysis for *dpsl* mRNA after treatment with 30 µM H_2_O_2._ B) Western blots for DPSL, 105 minutes after addition of 30 µM H_2_O_2_. Lanes 1–3 controls, 4–6 are from the three biological replicates used for the microarray and proteomics experiments. The polyclonal antibody to DPSL protein recognizes a background protein of slightly greater molecular weight than DPSL.

The aim of this study was to understand the oxidative stress interactome in *S. solfataricus*. The cells were grown until an OD_600_ reading of 1 was achieved, at which point oxidative stress was induced by the addition of H_2_O_2_ to a final concentration of 30 µM. This nonlethal concentration was selected based on previous studies from our group [9]. Both Northern and Western blot analyses previously demonstrated a significant up-regulation of a DPSL (SSO2079) in response to hydrogen peroxide. The response time of the *S. solfataricus* antioxidant defense network was determined using a time course of *dpsl* mRNA expression following H_2_O_2_ exposure (4–195 min). Northern blot analysis indicated that the DPSL gene transcript was strongly up-regulated in response to H_2_O_2_ stress (**Figure 1A**). This was confirmed at the protein level and found to be highly reproducible (**Figure 1B**). Using the *dpsl* gene as a hallmark for the oxidative stress response, microarray and 2D-DIGE experiments were designed to simultaneously evaluate changes in the *S. solfataricus* transcriptome and proteome following exposure to hydrogen peroxide. The transcriptional response was evaluated by microarray at 30 minutes post exposure to 30 µM H_2_O_2_ and the proteome was evaluated by 2D-DIGE at both 30 and 105 minutes post exposure to 30 µM H_2_O_2_.

### Microarray data

Changes in *S. solfataricus (P2*) gene expression were evaluated by single-channel analysis of expression arrays from NimbleGen. Results from the arrays indicated that the expression of 102 transcripts, out of 2,977 annotated *S. solfataricus* ORFs included on the array, significantly changed by at least 1.5 fold at 30 minutes post exposure to 30 µM H_2_O_2_ (**Supplemental Table S1**). The majority of these genes (73) were down-regulated, while 30 were at least 1.5 fold more abundant (Table 1). Results from the NimbleGen array were validated using a second microarray platform from Isogen Life Science (data not shown).

**Table 1:** *Sulfolobus solfataricus* transcriptionally regulated genes (for complete list see supplemental table S1).

Regulated gene	Gene ID	p value	Fold change	COG	Promotor
DPSL antioxidant protein	SSO2079	1.09E-08	41.53	COG2406	Y
Archaeal Rieske-type ferredoxin (arf)	SSO2080	2.17E-04	37.09	COG2146	N
Metal tion transporter, putative	SSO2078	7.52E-05	31.61	COG1914	Y
Membrane conserved hypothetical protein	SSO2568	6.75E-08	7.914	COG1814	N
Peroxiredoxin, bacterioferritin comigratory protein homolog (bcp-2)	SSO2121	1.89E-04	7.024	COG0450	Y
hypothetical protein	SSO2644	1.09E-06	6.728	COG1196	Y
Metal ion transporter, putative	SSO2076	2.17E-04	5.933	COG1914	Y
Glycerol-3-phosphate dehydrogenase chain C (anaerobic) (glpC)	SSO2643	6.26E-07	5.165	COG0247	Y
Ferric uptake regulation protein (fur) (Transcription regulator)	SSO2244	3.98E-06	4.254	COG0735	Y
hypothetical protein (glycerol 3-phosphate dehydrogenase)	SSO2645	5.38E-05	4.254		Y
Phosphoribosylformylglycinamidine cyclo-ligase (AIR synthetase) (AIRS) (purM)	SSO0636	1.57E-02	2.331	COG0150	Y
hypothetical protein (potenial transcription regulator SpoVT_AbrB_like DBD)	SSO2620	5.50E-04	2.215	COG0704	N
Conserved hypothetical protein (SufD-like)	SSO0928	2.24E-05	2.174	COG0719	N
hypothetical protein (Ferritin/ribonucleotide reductase like)	SSO2621	2.17E-04	2.081		Y
Glutamine synthetase (glutamate ammonia ligase) (GS). (glnA-1)	SSO0366	2.99E-03	1.942	COG0174	N
hypothetical protein	SSO2023	4.91E-03	1.872	COG0121	N
mRNA 3′-end processing factor, putative	SSO0761	1.34E-03	1.856	COG1782	N
Glutamine phosphoribosylpyrophosphate amidotransferase) (ATase) (GPAT) (purF-1)	SSO0632	8.02E-04	1.851	COG0034	Y
Phosphatase, putative (nagD-like)	SSO2355	3.41E-03	1.849	COG0647	Y
Conserved hypothetical protein	SSO2332	1.81E-02	1.827	COG0455	Y
Ammonium transporter	SSO1054	3.46E-02	1.794	COG0004	N
Conserved hypothetical protein (SufB-like)	SSO0927	1.33E-03	1.735	COG0719	N
ABC transporter, permease protein	SSO2671	1.61E-02	1.704	COG1173	Y
Conserved hypothetical protein	SSO1093	3.07E-02	1.68	COG1530	Y
Phosphoribosylamine–glycine ligase (GAR synthetase) (GARS) (purD)	SSO0635	4.28E-02	1.666	COG0151	Y
hypothetical protein	SSO1373	2.46E-03	1.659		
hypothetical protein	SSO3128	1.34E-03	1.629	COG1848	Y
Oxidoreductase	SSO3014	3.52E-02	1.563	COG0667	N
Conserved hypothetical protein (potenial transcription regulator SpoVT_AbrB_like DBD)	SSO0923	7.36E-04	1.546	COG0704	Y
Conserved hypothetical protein	SSO0046	2.74E-04	1.526	COG0084	Y
Oxidoreductase, putative	SSO2794	2.99E-04	−2	COG0437	Y
Pyruvate synthase delta chain (Pyruvic-ferredoxin oxidoreductase delta chain) (porD-1)	SSO7412	3.31E-02	−2.01	COG1144	Y
Conserved hypothetical protein	SSO1172	4.36E-02	−2.04	COG1449	N
Oxidoreductase, putative	SSO2795	1.38E-03	−2.06	COG0243	Y
Acetylornithine deacetylase (argE-2)	SSO1007	1.77E-05	−2.19	COG0624	Y
Conserved hypothetical protein	SSO1004	5.50E-04	−2.31	COG0277	Y
Conserved hypothetical protein	SSO1005	1.71E-03	−2.34		Y
Arabinose ABC transporter, arabinose binding protein	SSO3066	3.57E-05	−2.37	COG1653	N
Isocitrate lyase (aceA/icl)	SSO1333	9.01E-04	−2.37	COG2224	Y

The mRNAs coding for DPSL (SSO2079), and the two flanking genes (SSO2078 and 2080) showed the largest changes in abundance, 31 to 41 fold increase (**Table 1**). The genes on either side of *dpsl* are a hypothetical protein (SSO2078) and an archaeal Rieske-type ferredoxin (*arf*) (SS02080). Ferredoxins are iron-sulfur proteins that mediate a wide range of electron transfer reactions. The genomic neighborhood and up-regulation of SSO2080 in response to H_2_O_2_ suggests that this protein is involved in maintaining intracellular redox potentials. *dpsl* (SSO2079) and arf (SS02080) are adjacent genes transcribed from the same strand, while the hypothetical protein, SSO2078, is on the opposite strand. PSI-Blast [51], [52], conserved domain search [53] and COG analysis [54], [55] all suggest that SSO2078 is an inorganic ion transporter primarily responsible for Mn^2+^ and Fe^2+^ mobilization. The up-regulation of a metal transporter in response to oxidative stress is not unusual. In fact, an increase in intracellular iron is consistent with the H_2_O_2_ stress induced oxidation and subsequent liberation of iron from proteins with Fe-S centers. H_2_O_2_ mediated degradation of Fe-S clusters is further supported by the observed up-regulation of genes involved in Fe-S clusters biosynthesis. SSO0927 (*sufB*) and SSO0928 (*sufD*) are homologous to members of the bacterial Sulfur assimilation (SUF) operon, which is specifically adapted to synthesize Fe-S clusters when iron or sulfur metabolism is disrupted by iron starvation or oxidative stress [56].

A number of likely regulators of transcription were found in the microarray data. For example, the gene located directly upstream of the putative SUF operon, SSO0923, annotated as a conserved hypothetical protein is also up-regulated. This ORF contains a SpoVT_AbrB-like DNA binding domain (DBD) in the N-terminus. Transcription factors of the SpoVT_AbrB family typically share the highest sequence identity in the N-terminal DBD, while the C-terminal multimerization domains are less conserved [57]. The expression profile, genomic context, and the N-terminal SpoVT_AbrB-like DBD of SSO0923 suggest that this protein may be a transcriptional regulator of genes involved in iron and/or sulfur metabolism. A second gene containing a putative SpoVT_AbrB-like DNA binding domains SSO2620 was also up-regulated. Flanking this is SSO2621, which was also up-regulated, and according to InterPro (Integrated resource of Protein Families, Domains and Sites) has a Ferritin/ribonucleotide reductase-like signature in this sequence [58]. The presence of a ferritin-like 4-helix bundle is very suggestive of a role in iron sequestration and/or in metal dependent electron transfer.

The ferric uptake regulator (Fur), SSO2244, was also up-regulated. Fur has a well-established roll in oxidative stress response and functions as both an activator and a repressor [59], [60]. A single copy of this gene was found in the *S. solfataricus* genome, where as *B. subtilis* contains three Fur paralogs that coordinate gene expression in response to iron (Fur), zinc (Zur), or H_2_O_2_ (PerR). Interestingly, Fur regulates the expression of DPS in *Cyanobacterium Nostoc* PCC7120 [61] and a peroxiredoxin in *Cyanobacterium Synechocystis* [60]. Although the regulatory repertoire for Fur has not been mapped in *S. solfataricus,* the microarray data supplied an obvious list of genes to be tested.

### Proteomics data

**Figure 2:**
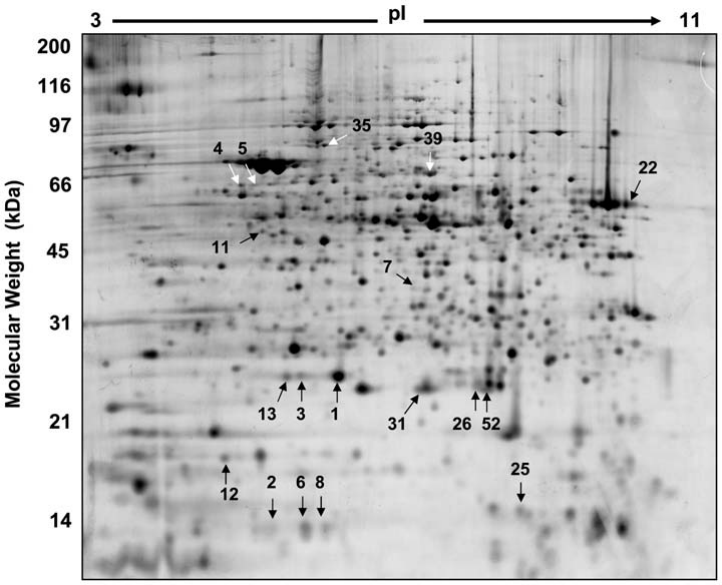
Regulated proteins of the *S. solfataricus* proteome after exposure to 30 µM H_2_O_2_. Approximately 818 spots (common to all gels) were used in the CyDye 2D DIGE analysis. 18 protein spots changed significantly in abundance 105 minutes post H_2_O_2_. Spots that changed in abundance are indicated with arrows. Protein identifications were made using in-gel proteolysis followed by LC-MS/MS and are listed in Table 2.

Hydrogen peroxide induced changes to the *S. solfataricus* proteome, including changes in protein abundance and post-translational modification (30 and 105 min post-H_2_O_2_) were investigated using CyDye based 2D-DIGE analysis. Greater than 1000 spots were found on each gel and after filtering to remove irregularities, 818 spots were used in the analysis across all gels (**Figure 2** **, Supplemental Table S2**). A single protein spot changed 30 minutes after H_2_O_2_ exposure and 29 after 105 minutes using a q-value (false discovery rate) cutoff of <0.04. At the later time point, nineteen were more abundant and 10 less abundant. Quantification of protein spots revealed changes ranging from −4.07 to +8.12 fold (**Table 2**).

**Table 2:** *Sulfolobus solfataricus* regulated proteins.

Regulated protein	Gene ID	Mass (Da)	pI	MS SCORE	pvalue	fdr	qvalue	Fold Difference	2D spot #	COG	Promotor
DPS-Like (Conserved hypothetical protein)§	SSO2079	21927	5.38	219	6.16E-06	5.04E-03	2.56E-03	8.12	1	COG2406	Y
DPS-Like (Conserved hypothetical protein)§	SSO2079	21927	5.38	233	1.33E-02	1.23E-01	6.25E-02	5.20	13	COG2406	Y
Small heat shock protein hsp20	SSO2427	20084	5.31	260	1.13E-02	1.31E-01	5.16E-02	4.92	3	COG0071	Y
DPS-Like (Conserved hypothetical protein)§	SSO2079	21927	5.38	86	1.13E-02	1.31E-01	5.16E-02	4.92	3	COG2406	Y
Thermosome alpha subunit (chaperonin alpha subunit)(thsA)	SSO0862	59695	5.35	491	4.45E-02	1.85E-01	9.39E-02	2.86	5	COG0459	Y
Thermosome beta subunit (chaperonin beta subunit) (thsB)	SSO0282	60387	5.56	501	4.45E-02	1.85E-01	9.39E-02	2.86	5	COG0459	Y
Thermosome alpha subunit (chaperonin alpha subunit)(thsA)	SSO0862	59690	5.35	283	2.51E-02	1.60E-01	8.16E-02	2.52	4	COG0459	Y
Oxidoreductase (putative)	SSO2588	15686	7.74	172	1.27E-03	6.29E-02	3.20E-02	2.35	25	COG0517	N
Transcriptional regulator, marR family, putative	SSO1082	18313	9.12	104	1.27E-03	6.29E-02	3.20E-02	2.35	25	COG1846	Y
Small nuclear ribonucleoprotein (snRNP) homolog (putative)	SSO0276	16504	7.82	163	1.27E-03	6.29E-02	3.20E-02	2.35	25	COG1958	Y
Translation elongation factor EF-1alpha *	SSO0216	48573	8.93	540	3.00E-02	1.64E-01	8.32E-02	2.03	22	COG0050	Y
Peroxiredoxin (putative)§	SSO2121	24786	6.85	119	2.21E-02	1.50E-01	7.61E-02	1.93	26	COG0450	Y
Superoxide dismutase [Fe] (sod)	SSO0316	24228	6.71	144	2.21E-02	1.50E-01	7.61E-02	1.93	26	COG0605	Y
gamma-glutamyltranspeptidase, hypothetical	SSO3216	53136	5.67	75	6.78E-02	2.34E-01	1.19E-01	1.89	12	COG0405	Y
Peroxiredoxin (putative)§	SSO2121	24786	6.85	98	9.96E-05	1.63E-02	8.29E-03	1.87	31	COG0450	Y
Rubrerythrin (rr)	SSO2642	16081	5.44	134	1.43E-03	6.29E-02	3.20E-02	1.79	6	COG1592	Y
Superoxide dismutase [Fe] (sod)	SSO0316	24228	6.71	163	4.59E-03	8.91E-02	4.53E-02	1.70	52	COG0605	N
Glutamyl-tRNA(Gln) amidotransferase subunit E	SSO0936	71661	5.91	161	5.08E-04	5.19E-02	2.64E-02	−1.61	35	COG2511	Y
NAD specific glutamate dehydrogenase (gdhA-4) * §	SSO2044	46091	6.5	436	9.44E-04	5.93E-02	3.02E-02	−2.13	39	COG0334	N
Signal recognition particle receptor protein	SSO0348	40390	5.29	237	1.83E-03	6.51E-02	3.31E-02	−2.84	11	COG0552	Y
X-Pro aminopeptidase (putative)	SSO0010	40594	5.35	141	1.83E-03	6.51E-02	3.31E-02	−2.84	11	COG0006	Y
Rubrerythrin (rr)	SSO2642	16081	5.44	118	2.95E-02	1.64E-01	8.32E-02	−3.06	8	COG1592	Y
Rubrerythrin (rr)	SSO2642	16081	5.44	277	2.02E-02	1.47E-01	7.45E-02	−3.83	2	COG1592	Y
Ribose phosphate pyrophosphokinase	SSO1045	32517	6.13	161	5.89E-05	1.20E-02	6.13E-03	−4.07	7	COG0462	N

* Proteins found among the phosphorylated proteins.

§ proteins found among microarray and proteomics regulated genes.

Genes promotor based on *Sulfolobus solfataricus* P2 Genome TIGR website, http://cmr.jcvi.org/tigr-scripts/CMR/GenomePage.cgi?database=ntss02.

Proteins showing altered abundance were identified from the gel spots using in-gel proteolysis and LCMS/MS analysis. Peptides were queried against an expanded in-house database using MASCOT. Previous work from our lab has demonstrated that the annotated ORFs at TIGR and NCBI do not provide complete coverage of all translated regions on the *S. solfataricus* chromosome [43] so, our in-house database includes all ORFs greater than 50 amino acids. Protein MOWSE scores ranged from 75 to 540 using only peptide scores >32 (p<0.05). The validity of all assigned MS/MS spectra used for identification of regulated proteins was confirmed by manual inspection. A complete list of the regulated proteins, mass spectrometry scores, molecular weights, pI, fold change of abundance and annotation is shown in **Table 2** (**Supplemental Table S3** includes active links). Based on the clusters of orthologous groups (COG) partitioning, the regulated proteins were from 18 of the 26 groups. Functional groups include; translation, transcription, amino acid transport and metabolism, lipid metabolism, amino acid biosynthesis, posttranslational modification, energy production and conversion, inorganic ion transport and metabolism, and importantly, antioxidant and cellular detoxification.

The single largest fold change in the proteomic analysis was for DPSL. This finding was consistent with the microarray, northern, and western analyses (**Figure 1**). The DPSL protein was found in three different gel spots that all increased in abundance at 105 min post H_2_O_2_ exposure, indicating PTM. The three isoforms of this protein differed in pI and in protein abundance. The isoform with the highest pI is ∼8.5 times more abundant following H_2_O_2_ exposure (**Table 2** **and** **Figures 2** **&** **3**, spots 1, 3, and 13). The specific location and type of PTMs on the DPSL protein has yet to be identified, but the change in pI suggests a modification that alters side chain charge. The robust up regulation, demonstrated ability to reduce H_2_O_2_ [9], and absence of catalase in the genome of *S. solfataricus* [62] suggest that DPSL has an important role in managing oxidative stress.

**Figure 3:**
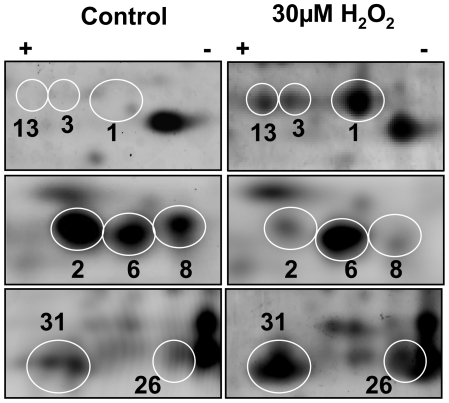
Post translational modification is a common feature in *S. solfataricus*. Three of the proteins that are regulated after H_2_O_2_ treatment are known to be important in oxidative stress and were found in multiple gel spots. Modifications can alter the pI and MW position on 2D gels. The paired panels show close ups of SyproRuby stained 2D gels, 105 minutes after addition of 30 µM H_2_O_2_. Top, DPSL (SSO2079; 21639 Da; pI 5.25) spots 1, 3 and 13. Middle, Rubrerythrin (SSO2642; 16081 Da; pI 5.44) spots 2, 6 and 8. Bottom, Peroxiredoxin (SSO2121; 24786Da; pI 6.85) spots 26 and 31.

Rubrerythrin (SSO2642) had a complex pattern of regulation This protein is found in many air-sensitive bacteria and archaea [63], [64] and recent data has demonstrated a role in anaerobic detoxification where is catalyzes the reduction rather than disproportionation of superoxide and hydrogen peroxides [65]. Rubrerythrin was up-regulated on microarray and present in three spots (isoforms) on both the 30 and 105-minute 2D gels. These spots (2, 6 and 8, **Figures 2** **and** **3** **, and** **Table 2**) had different pI's and different patterns of expression. For example, the isoform in spot 6 increased significantly in abundance in stressed cells, whereas spots 2 and 8 decreased in abundance by 3.8 fold. This is another example where PTM plays a role in differential abundance of a protein. This observation highlights one of the strengths of the 2D-DIGE method as a tool for studying protein expression and proteome-wide PTM.

Rubrerythrin is involved with cellular redox potential in other organisms as well. For example, it was up-regulated in *Methanothermobacter thermautotrophicus* by H_2_O_2_ along with other redox enzymes [26]. *In vitro* analysis of rubrerythrin from *D. vulgaris* and *Clostridium perfringens* found NADH peroxidase activity as part of a novel oxidative stress protection system found in these organisms [27], [29], [66]. In the obligate anaerobe *Porphyromonas gingivalis,* which lacks catalase, rubrerythrin expression increased in response to H_2_O_2_ stress [28], [67] while a knock out mutant did not survive. *P. furiosus* rubrerythrin, which was the first to be characterized from an archaeal hyperthermophile, functions in an NADH-dependent, hydrogen peroxide:rubredoxin oxidoreductase peroxidase system [65]. The specific mechanism at work here remains to be elucidated, but the identification of different isoforms represents a starting point for such studies. *S. solfataricu*s rubrerythrin is also part of a larger cluster of genes that are all up-regulated. The gene directly downstream (SS02645) is annotated as a glycerol-3-phosphate dehydrogenase (*glpC*). GLPC is an NADH-dependent enzyme that catalyzes the oxidation of glycerol 3-phosphate to dihydroxyacetone phosphate, the first step in glycerol synthesis, and has been shown to be important for adaptation to diverse environmental perturbations in *Saccharomyces cerevisiae* [68]. Although it is reasonable to assume a similar role for this protein in *S. solfataricus*, it is worth nothing that sequence analysis indicates the protein contains an additional heterodisulfide reductase domain. The conspicuous genomic location of *glpC*, directly downstream of a rubrerythrin, and the extra domain is very suggestive of an alternative function.

Peroxiredoxin (SSO2121), also known as Bcp2, is another regulated protein with ties to oxidative stress response. It is up-regulated in the microarray at 30 min post H_2_O_2_ exposure (**Table 1**) and is also up-regulated at the 30 and 105-minute time points at the protein level (**Table 2** **and** **Figure 3**). Peroxiredoxin is a thioredoxin-dependent protein that plays an important role in the peroxide-scavenging system in *S. solfataricus* [69], [70] and has been shown to protect chromosomal DNA from nicking by metal-catalyzed oxidation. Peroxiredoxin homologs are prevalent in thermophiles and *S. solfataricus* codes for four orthologs: Bcp1 (SSO2071), Bcp2 (SSO2121), Bcp3 (SSO225) and Bcp4 (SSO2613) [62]. It has been proposed that the Bcps represents a constitutive antioxidant system using Bcp1 and Bcp4 to prevent endogenous peroxide accumulation [37], while Bcp2 and Bcp3 are induced in response to external peroxides, which in the case of Bcp2, is consistent with our data.

Two of the regulated proteins (SSO1098 and SSO2588) were listed as hypothetical. Sequence comparison and structural prediction failed to provide any significant clues to the cellular role of SSO1098. Protein SSO2588, on the other hand, turned out to be very interesting. It has 61% sequence similarity to an oxidoreductase in *Sulfolobus tokodaii* (ST2348). Oxidoreductases perform a variety of functions including chaperones for protein folding, renaturation, degradation, electron transport, and participate in the response to oxidative stress [71]. SSO2588 also has sequence similarity with three other genes in *S. solfataricus* (SSO1075, SSO3174, and SSO3230), though none of these were differentially regulated in response to H_2_O_2_. An independent study that looked only at disulfide oxidoreductases, reported the induction of SSO0192 from *S. solfataricus* after exposure to higher H_2_O_2_ dosage [72]. The presence of additional putative members of this protein family that were not regulated in this study_,_ suggests that *S. solfataricus* has developed specialized roles for this important class of protein, or that it is regulated only at the protein level and is present in few copies per cell.

A number of the regulated proteins like superoxide dismutase, heat shock protein, peroxiredoxin and elongation factor-1 alpha found in this study are consistent with a general oxidative stress response [24], [73], [74]. Homologues to superoxide dismutase (SOD) (such as SSO0316), participate in the scavenging of highly reactive oxygen species across all domains [75], [76]. Since SODs catalyze the production of H_2_O_2_, the reason for activation here is not entirely clear, but it may be part of the general oxidative stress response network. Another important protein for general oxidative stress is Translation Elongation Factor 1A (eEF1A), which is also up-regulated in this study (SSO0216). In the H9c2 rat embryonic cell line, EF-1α protein levels undergo rapid increase upon treatment with H_2_O_2_ [77]. It is worth noting that mouse eEF1A-2 interacts with peroxiredoxin-I (Prdx-I) in protecting cells from oxidative stress induced apoptosis. Mouse cells transfected with both eEF1A-2 and Prdx-I have increased resistance to peroxide-induced cell death compared to single transfectants [78]. The homologues for both of these eukaryotic proteins were up-regulated in our study, which implies that a similar defense mechanism may be present in *S. solfataricus*.

Beyond changes in mRNA and protein abundance, PTM modifications (e.g. phosphorylation, sulfation, glycosylation, carbonylation and cysteine oxidation) are important regulators of protein activity. As discussed above with respect to DPSL and rubrerythrin, these modifications can alter the charge of a protein, which will shift the position on a 2D gel [73]. Of the 19 regulated proteins found in this study, 5 were identified in more than one spot; DPSL (SSO2079), superoxide dismutase (SSO0316), peroxiredoxin (SSO2121), rubrerythrin (SSO2642), elongation factor 1-alpha (SSO0216) and thermosome alpha subunit (SSO0862). Only two of these proteins were regulated at the level of mRNA, however, based on their known functions and the 2D gel data, it seems likely each of them acts via a mechanism controlled by PTM. In addition to enzyme catalyzed covalent modifications, direct chemically induced changes can occur. For example, the active site cysteine residue of peroxiredoxin can be oxidized to cysteic acid [79]. This conversion adds a negative charge to the protein and may explain why peroxiredoxin was in multiple spots. Interestingly, analysis of SSO2121 peroxiredoxin using ProMoST (Protein Modification Screening Tool; http://proteomics.mcw.edu/promost) indicates that modification of one cysteine to cysteic acid would shift the position horizontally one pH unit. This predicted shift precisely matches the observed shift of gel spot 31 (**Figures 2** **and** **3**).

**Figure 4:**
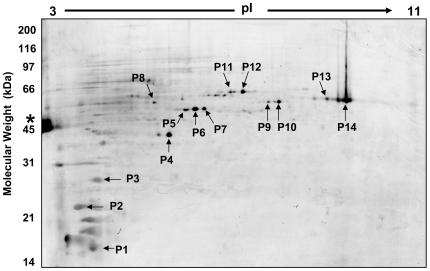
2D gel of the *S. solfataricus* phospho-proteome 105 minutes after H_2_O_2_ treatment. The gel was stained with phosphoprotein specific stain ProQ Diamond. Each of the numbered spots was picked and the proteins were identified using in-gel proteolysis followed LC-MS/MS. Ovalbumin (* on left) is a 45 kDa phosphoprotein standard.

Phosphorylation has important roles in regulation and signal transduction in bacteria and eukarya. Recent evidence has implicated H_2_O_2_ itself as an intracellular messenger that modulates the phosphorylation of serine, thereonine and tyrosine residues [80]–[82]. Although protein kinases are prevalent in all three domains of life, relatively little is known about the use of phosphorylation in archaea. The addition or removal of a phosphate group alters protein pI, resulting in a horizontal shift on a 2D gel. The ability to globally screen for PTMs based on gel shift is a major advantage of the 2D DIGE approach. To take advantage of this, the fluorescent phosphoprotein specific stain, Pro-Q Diamond^®^ was used [83]. Analysis of control and H_2_O_2_ treated samples on 2D gels showed clear changes in the pattern of phosphorylation (**Figure 4**). Spots containing high levels of phosphorylation were selected for in-gel digestion and protein IDs were made from 14 of them (**Table 3**). Ten of the 14 spots contained more than one protein; therefore, the specific phosphoprotein could not always be determined. Twenty different proteins were identified from the 14 spots, 7 of which were found in more than one horizontally separated position as would be expected for differential phosphorylation. Three of the phospho-stained proteins (S-adenosylmethionine synthetase, Glutamate dehydrogenase, and elongation factor-1 alpha) were among the differentially expressed proteins listed in **Table 2**. The detailed characterization of specific protein phosphorylation has only been described for a few *Sulfolobu*s proteins [84]–[86]. In general, we noticed less phosphoprotein staining for *S. solfataricus* in comparison to the Bacteria *Mycoplasma penetrans* and *Bacillus subtilis*, Chang's human liver cells, mouse Oocytes and *Leishmania donovani* [87]–[91]. This analysis clearly shows that the state of protein phosphorylation in *S. solfataricus* is dynamic and the identification of phosphopeptides using enrichment techniques and more sensitive mass analysis will be undertaken in the future.

**Table 3:** Protein Identification from phospho-stained spots.

NAME	Gene ID	Mass (Da)	pI	MS Score	2D spot #	COG
ribosomal protein L12	SSO0342	11284	4.84	178	P1	COG2058
SSU ribosomal protein S19E	SSO0353	18107	10.03	155	P1	
ribosomal protein L15	SSO0696	16187	10.45	135	P1	COG0200
ribosomal protein S4	SSO0073	20735	9.89	258	P2	COG0522
Phosphohistidine phosphatase (SixA)	SSO1195	18169	5.52	100	P2	COG2062
Disulfide oxidoreductase	SSO0192	25902	4.7	394	P3	COG0526
30S ribosomal protein S3AE	SSO0746	23585	9.71	138	P3	
Succinyl-CoA synthetase, beta subunit	SSO2483	37388	5.57	512	P4	COG0045
hypothetical protein	SSO0286	42681	5.78	298	P5	COG1980
Thermostable Carboxypeptidase (cpsA-1)	SSO1355	43326	5.93	218,419,183	P5,P6,P7	COG1473
Thermostable carboxypeptidase (cpsA-2)	SSO1952	43250	5.93	143,301,150	P5,P6,P7	COG1473
Adenylosuccinate synthase (IMP aspartate ligase)	SSO0242	37417	5.83	206, 94	P6, P7	COG0104
Glutamate dehydrogenase (gdhA-4) *↓	SSO2044	46091	6.5	157,278	P7, P10	COG0334
Long-chain-fatty-acid-CoA ligase (fadD-1)	SSO0369	51587	6.11	163	P8	COG0318
Hypothetical protein	SSO2635	52481	5.6	741	P8	COG0709
S-adenosylmethionine synthetase *↑	SSO0199	45382	5.86	680	P9	COG1812
Conserved hypothetical protein	SSO1389	43073	6.13	55	P9	COG1517
serine hydroxymethyltransferase	SSO0530	48535	6.22	261,463	P11, P12	COG0112
S-adenosylhomocysteine hydrolase	SSO0755	45936	6.3	146, 189	P11, P12	COG0499
Elongation factor 1-alpha *↑	SSO0216	48573	8.93	573,104	P13, P14	COG5256

* Found among the regulated proteins, ▒ arrow indicates status of regulation

### Chemical tagging of Redox reporter proteins

**Figure 5:**
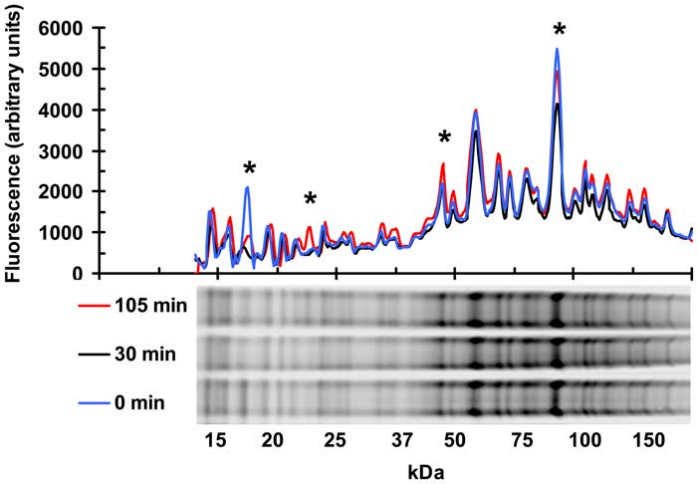
Protein thiol reactivity changes in H_2_O_2_ stressed *S. solfataricus*. Proteome-wide labeling of free cysteine thiols, with BODIPY maleimide, shows that there is a population of redox sensitive proteins. Plot on the top shows the average fluorescent signal with respect to molecular weight. The fluorescent signal from three experiments was combined and normalized for total protein. The gel lanes at the bottom show the actual data form one experiment; with 0 (*blue line*), 30 (*black line*) and 105 minute (*red line*) samples. * indicates protein bands that were highly sensitive to changes in redox potential.

The redox state of cysteine residues in many proteins are sensitive to the overall redox potential within a cell and the reactivity of individual protein thiols to oxidation can be part of signal transduction pathways [92]. For this reason, we were interested in identifying *S. solfataricus* proteins that may be redox sensors. It was reasoned that these proteins could be detected by differences in cysteine reactivity under different redox potentials. Measuring the oxidation state of proteins *in vivo* is challenging because many of the free cysteines are inaccessible to reagents under non-denaturing conditions and the surface-exposed thiols are not necessarily preferred targets for oxidative stress-mediated modifications. We developed and tested a protocol that consistently labeled reduced cysteine side chains under native and denaturing conditions using the fluorescent dye BODIPY^®^ 577/618 maleimide. Comparison of whole proteome labeling patterns before and after treatment with H_2_O_2_ revealed a limited number of proteins that were highly sensitive to the redox potential (**Figure 5**). The curves show the average fluorescence intensity after normalization for protein concentration from three separate experiments. The percentage of reduced-thiols across the proteome decreased 30 minutes after H_2_O_2_ exposure (black line). By the second time point (105 minutes, red line) there was a general recovery and some protein bands were more reactive than before treatment, suggesting that there may even be a slight over compensation or lower than normal oxidation potential in the cells when the stress pathways are in full operation. Future studies will be directed at identifying the specific proteins and sites used as redox sensors.

Integration of microarray and proteomics data presents a more complete biological perspective than either alone, however, in other studies where both mRNA and protein abundance have been examined, there has often been poor correlation between the two data sets [93]–[96]. In this study, DPSL was at the top of both lists for regulated mRNAs and proteins. The next three most up-regulated mRNAs (37, 32, and 8 fold) were not found to be regulated at the protein level. This can be explained because two of these are membrane proteins (SSO2078 and SSO2568) and the third codes for a protein of ∼10 kDa (SSO2080), all of which would not be expected to behave well in the 2D gel procedure. One of the four genes that appear on both lists is peroxiredoxin, which as discussed above is known to play a role in oxidative stress. The third common gene is NAD specific glutamate dehydrogenase (SSO2044), which is down-regulated in both types of analysis (**Table 2** **and Supplemental Table S1**). It is a member of the oxidoreductase superfamily, which was discussed in relation to oxidative stress earlier. In addition to SSO2044, we found three other regulated genes (SSO0632, SSO0684, and SSO0936) which participate in the glutamate metabolism pathway. This could represent a good example of gene regulation on the level of a pathway. Glutamate metabolism is one of the central cellular pathways in *Sulfolobus* (http://www.genome.jp/dbget-bin/show_pathway?sso00251SSO2044) and is integrated with numerous other pathways such as glutathione metabolism, a major contributor to antioxidant protection. Gamma-glutamyltranspeptidase (SSO3216), a key enzyme in the glutathione metabolism, increases in abundance following oxidative challenge. Although Archaea are thought not to have glutamate cysteine ligase (GshA) or glutathione synthase (GshB), key enzymes in glutathione synthesis, there is a report of a putative gamma-glutamylcysteine ligase (GshA) from the archaea *Methanosphaera stadtmanae* [97]. The protein is similar to glutamatecysteine ligase and the bifunctional glutamate-cysteine ligase/glutathione synthetase that are involved in the first step of glutathione biosynthesis in many bacterial organisms [98], [99]. Using the GshA sequence form *M. stadtmanae* to search the *S. solfataricus* genome we identified a gene (SSO2815) with low, but significant sequence similarity (43% similarity, 23% identity) and could indicate the existence of glutathione-like system in Archaea.

In an attempt to expand the analysis beyond single or clusters of regulated proteins and mRNAs, the **D**atabase for **A**nnotation, **V**isualization and **I**ntegrated **D**iscovery (**DAVID**, NCBI) was used. DAVID showed that 25 out of 102 regulated mRNAs and 7 of the 24 regulated proteins have oxidoreductase activity. Both of these represent highly significant functional category enrichments. DAVID analysis also revealed that 19 of the regulated mRNAs are associated with transport and 18 of these are associated with membranes. The 2D-DIGE approach that was undertaken here was designed to maximize proteome coverage and was therefore biased toward soluble proteins. Another source of discrepancy between the methods is that transcriptome analysis is relatively good at measuring mRNAs at low copy number. Detection and quantitation of low abundance proteins is more difficult, regardless of the specific approach. With this in mind, it may not be surprising that only 3 of the regulated proteins (12%) came back as hypothetical compared with 26% of the regulated mRNAs. Both of these values are well below 50%, which represents the predicted ORF's in *S. solfataricus* that remain un-annotated. This suggests that the genes involved with oxidative stress response are more highly conserved across domains than archaeal genes in general.

**Figure 6:**
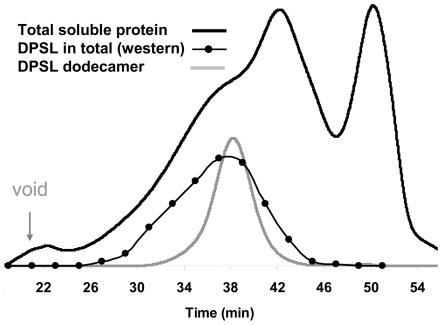
Size exclusion chromatography of DPSL. SEC data shows that a significant portion of DPSL from H_2_O_2_ stressed cells is part of a larger molecular complex. Total soluble protein, 105 min. after H_2_O_2_ exposure (black line) and purified recombinant DPSL separated under identical conditions (gray line) were detected by monitoring at 280 nm. Western blot analysis of the total soluble protein fractions using anti-DPSL antibody shows that *in vivo* part of the DPSL elutes earlier (27–34 min) in comparison to the purified wild type DPSL (∼38 min) indicating that it is part of a larger molecular complex.

DPSL regulation was the most unifying feature between the two “omics” data sets. To better understand the role of this dodecameric protein complex in the oxidative stress response, *S. solfataricus* was exposed to H_2_O_2_ and after 105 minutes total soluble protein was analyzed by size exclusion chromatography. The elution profile of the total soluble protein was distributed across the limits of the Superose-6 column (**Figure 6**). A Western-blot analysis, using a polyclonal antibody to DPSL, was used to compare the elution profile of DPSL in vivo with purified recombinant dodecameric protein. DPSL from H_2_O_2_ stressed cells had a prominent shoulder which eluted earlier, indicating that a portion of the protein cage was part of a larger complex. Based on 1D SDS-PAGE a number of proteins were present in fractions containing the DPSL complex (27–34 min), so it was not possible to distinguish between co-eluting proteins and potential DPSL interaction partners. To address this, purified recombinant dodecameric DPSL was immobilized onto an amino-link resin, creating an affinity column. *S. solfataricus* cell lysate was incubated with the immobilized DPSL followed by several steps of washing to remove proteins that bound nonspecifically. After extensive washing, proteins were eluted using pH 2.5 glycine or trypsin was added directly to a small aliquot of DPSL beads. Control experiments were conducted in parallel using deactivated amino-link beads to test for proteins that interacted nonspecifically with the resin. LCMS based peptide sequencing revealed superoxide dismutase (SSO0316) and peroxiredoxin (SSO2121) as having high affinity for DPSL. Both of these proteins were identified in the low pH elution and samples in which the bound material was digested directly off the beads after washing, in three replicate experiments and were not detected in the controls. Superoxide dismutase and peroxiredoxin were both up-regulated in the 2D-DIGE experiment and the later was also on the microarray list. This strongly suggests that the oxidative stress response leads to assembly of a protein complex containing multiple catalytic capabilities. SOD removes the highly reactive superoxide radical producing H_2_O_2_ and molecular oxygen. DPSL and peroxiredoxin both scavenge peroxide, using metal and cysteine based mechanisms respectively. Based on the known reactions, it is straightforward to envision how a molecular machine containing all three enzymes could function efficiently in the removal of ROS. Such a molecular complex could also explain why SOD protein increased in abundance. Formation of this complex may slow protein turnover, leading to an increase in abundance without a change at the mRNA level. This also explains why a protein that produces H_2_O_2_ would appear up regulated in the proteomics experiments.

**Figure 7:**
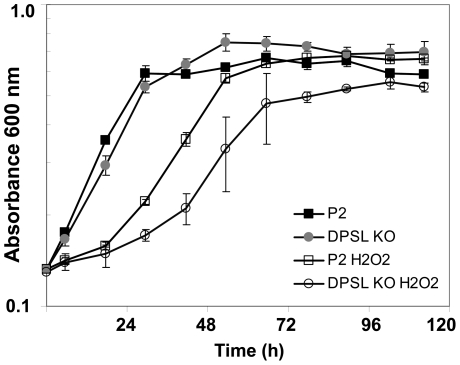
DPSL deficient strain of *S. solfataricus* is more sensitive to H_2_O_2_. *S. solfataricus* (P2) and a mutant lacking DPSL (DPSL KO) were cultured with and without 30 µM H_2_O_2_. P2 (solid square), P2 with H_2_O_2_ (open square), DPSL KO (gray circle), DPSL KO with H_2_O_2_ (open circle) n = 3.

How central is DPSL to the H_2_O_2_ response of *S. solfataricus*? Two possible scenarios are; DPSL is a central node for orchestrating the protective response and without it, sensitivity to oxidative stress dramatically increases, or DPSL is one member of a network with built in redundancy, and the loss of any one protein is not lethal. To test this, DPSL was inactivated by insertion of LacS into the coding sequence [35], [40]. Disruption of the gene was confirmed by DNA sequencing and DPSL inactivation was also checked by both PCR and western blot analyses (**Supplemental Figure S1**). Cells lacking DPSL had a significant lag in growth after exposure to H_2_O_2_ (**Figure 7**). However, this did not lead to large-scale cell death and the cultures eventually recovered, indicating that redundant pathways or compensating mechanisms exist to deal with H_2_O_2_ induced oxidative stress. Redundant or compensatory mechanisms have also been shown to be present in bacteria and eukaryotes, indicating a degree of similarity between the three domains of life.

**Figure 8:**
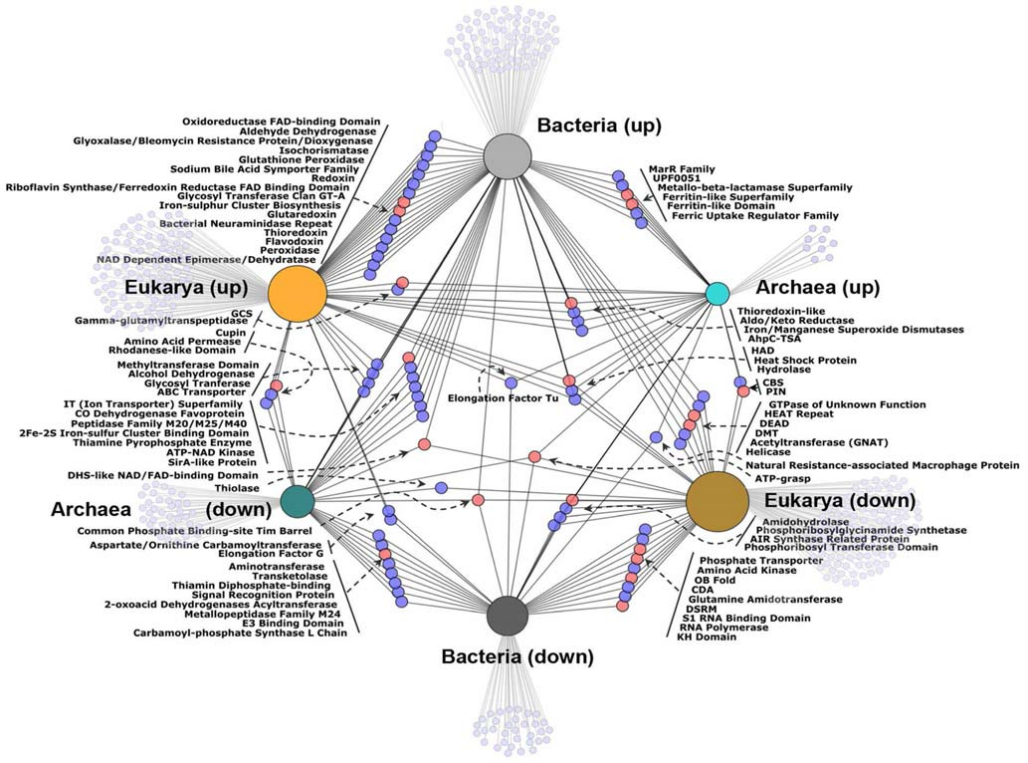
Network of shared mechanisms for oxidative stress response between Archaea, Bacteria, and Eukaryotes. Data from transcriptomics and proteomics experiments on *S. solfataricus*, *Bacillus*, *E. coli, and* Yeast after H_2_O_2_ exposure were combined to assess the relatedness of representative organisms across the three domains of life. Blue nodes represent protein families and salmon nodes represent protein clans. Smaller gray nodes show pfams unique to a particular domain and direction of regulation. The size of the node for each domain is scaled to according to number of regulated pfams.

The extensive data sets developed in this study for *S. solfataricus*, made it possible to evaluate the mechanisms and pathways that respond to H_2_O_2_ across all three domains of life. A composite analysis was made using the data presented here and that from previously published transcriptomics and proteomics experiments for Eukarya [13] and Bacteria [19], [23]. Data sets were converted into a congruent format by replacing official gene symbols, locus tags, or GI accession numbers, with Entrez Gene IDs. Once the gene lists were in a common format, the DAVID functional annotation tool was used to search for COGs, Pfams, and Gene Ontology to construct an interaction network. The combined list of 712 proteins representing 437 pfams was transferred to Cytoscape [48], [49] to create a graphical representation of the interaction network (Figure 8) This network was based on pfams to avoid specific differences in annotation between the three domains, which allowed the majority of regulated genes (83%) to be included in the analysis. Only three pfams were up-regulated in all three domains (superoxide dismutases, aldo/keto reductases, and thioredoxin-like) representing 3, 10, and 18 proteins from archaea, bacteria, and eukarya respectively (**Figure 8** **and Supplemental Table S4**). The node sizes for up and down-regulation in **Figure 8** are scaled to show the relative numbers of regulated genes. Although the studies used similar methods and concentrations of H_2_O_2_, a significantly larger number of regulated genes and proteins were detected in eukaryotes, reflecting their larger genomes and greater complexity. Three-way connectivity, with respect to up and down regulation, between domains in the network is limited, however, the majority of pfams were found in two of the three domains. For example, only 20% of the archaeal, and 25% of the bacterial pfams were unique (not connected by a node). For eukaryotes the number of unique (unconnected) pfams was higher (41%), again suggesting a more complex system. As a whole, the network indicates that oxidative stress response mechanisms are largely conserved across the three domains of life, although the specific details can be different. From an evolutionary stand-point, eukaryotes have much in common with prokaryotes, although many of the commonalities are not shared between archaea and bacteria, suggesting separate lateral transfer events [100] or adaptation to different environments.

### Conclusions

**Figure 9:**
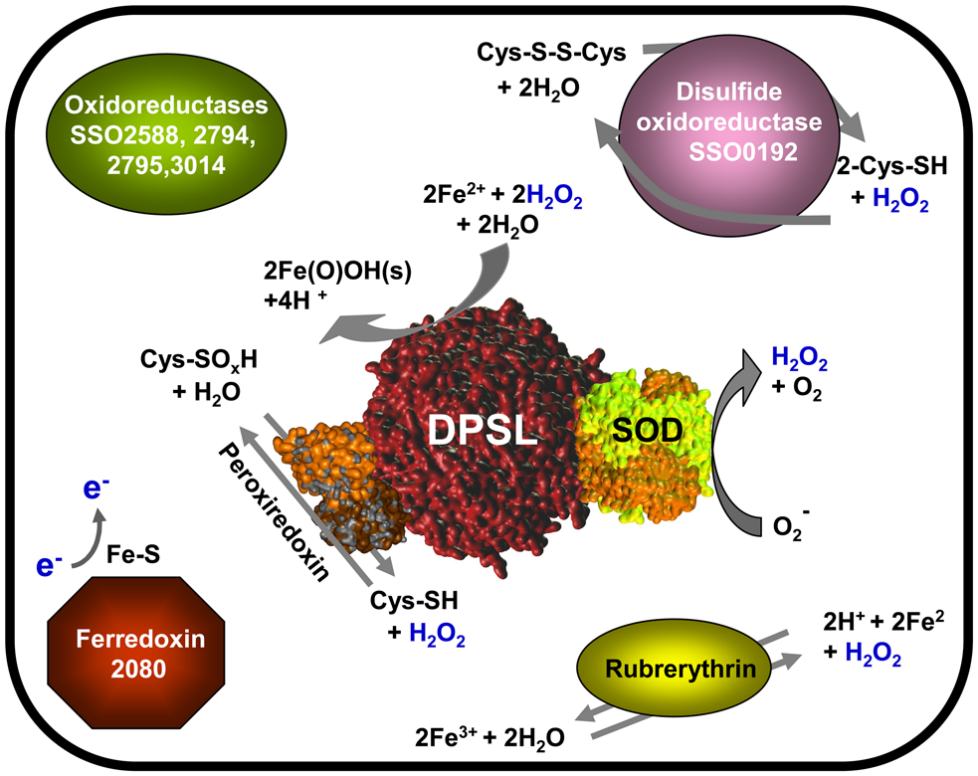
Schematic of the oxidative stress response in *S. solfataricus* based on mRNA and protein regulation after hydrogen peroxide exposure. Numbers indicate gene number (SSO). DPSL, SOD, and Peroxiredoxin are part of a molecular complex that can coordinate removal of ROS by converting highly reactive superoxide into H_2_O_2_ and then using this as substrate in subsequent reactions.

A large body of data exists on oxidative stress in eukaryotes and bacteria. This study presents the first “omics” data for an archeal organism, *S. solfataricus*. Changes to the transcriptome, proteome, and global protein redox state were investigated affording a system-wide analysis. Based on this work, it is clear that regulation occurs at the level of mRNA abundance, protein abundance, and PTM. The cellular response is mounted most strongly through DPSL, but includes a diverse set of coordinated mechanisms. The stress related proteins SOD, peroxiredoxin, rubrerythrin, and heat shock were regulated, supporting this idea. Significantly, it was shown that a portion of the cellular DPSL protein pool is present in a complex likely to include SOD and peroxiredoxin (**Figure 9**). The catalytic mechanisms of these three proteins integrate nicely and to our knowledge this is the first report of a supramolecular complex that could coordinate removal of ROS. Overall this complex is reminiscent of the recently described stressosome which also assembles around a protein cage [101]. The stressosome, however, functions as a signaling hub, where as the complex presented here is better described as a processing center for ROS. The transcriptomics and proteomics data presented here, together with the biochemical characterization of DPSL place this ferritin-like protein cage at the center of a cellular oxidative stress scheme (**Figure 9**). Even so, loss of DPSL is not catastrophic to *S. solfataricus* cells, suggesting crosstalk and redundancy in the response to oxidative stress. Crosstalk and redundancy are common in the bacterial and eukaryotic organisms used in the composite network analysis, which can serve as a starting point for making connections of similar mechanisms used by evolutionarily distant organisms. Much remains to be learned about the ferritin-like DPSL proteins, how they function *in vivo*, and the specific composition and role of larger complexes involved with ROS. Further studies of oxidative stress in archaeal organisms will undoubtedly help us to understand how organisms adapt to extreme environments, the evolution of mechanisms that combat oxidative damage, and could lead to novel therapeutic or prophylactic approaches.

## Supporting Information

Figure S1 LacS disruption mutant of the ssdpsl gene in S. solfataricus. A. PCR amplification of the dpsl gene from genomic DNA isolated from, Lane 2) S. solfataricus, strain P2; Lane 3) S. solfataricus strain 98/2; Lane 4) lacS insertion into the S. solfataricus strain 98/2 dpsl gene. DNA sequencing identified a single nucleotide difference between the S. solfataricus P2 and 98/2 dpsl genes. B. Western Blot performed on wild type and dpsl mutant cells stressed with 0, 20, 25 and 30 µM H2O2. Approximately 8 µg of protein was loaded in each lane and electrophoretically separated on a 15% SDS-polyacrylamide gel. Proteins were transferred to a nitrocellulose membrane and probed with polyclonal antibodies raised against purified recombinant SsDPSL protein. Star indicates the 22kDa SsDPSL induced protein.
(0.90 MB TIF)

Table S1 Sulfolobus Solfataricus regulated genes
(0.07 MB XLS)

Table S2 Statistical analysis for the 818 2D spots
(0.12 MB XLS)

Table S3 Sulfolobus solfataricus regulated proteins
(0.04 MB XLS)

Table S4 Proteins identified within Up-regulated Cytoscape network
(0.03 MB XLS)
