# Supplementary figures and images for: Something Old, Something New, Something Borrowed; How the Thermoacidophilic Archaeon Sulfolobus solfataricus Responds to Oxidative Stress
Source: PLoS One. 2009 Sep 16;4(9):e6964. doi: 10.1371/journal.pone.0006964 (PMC2739297; doi:10.1371/journal.pone.0006964)

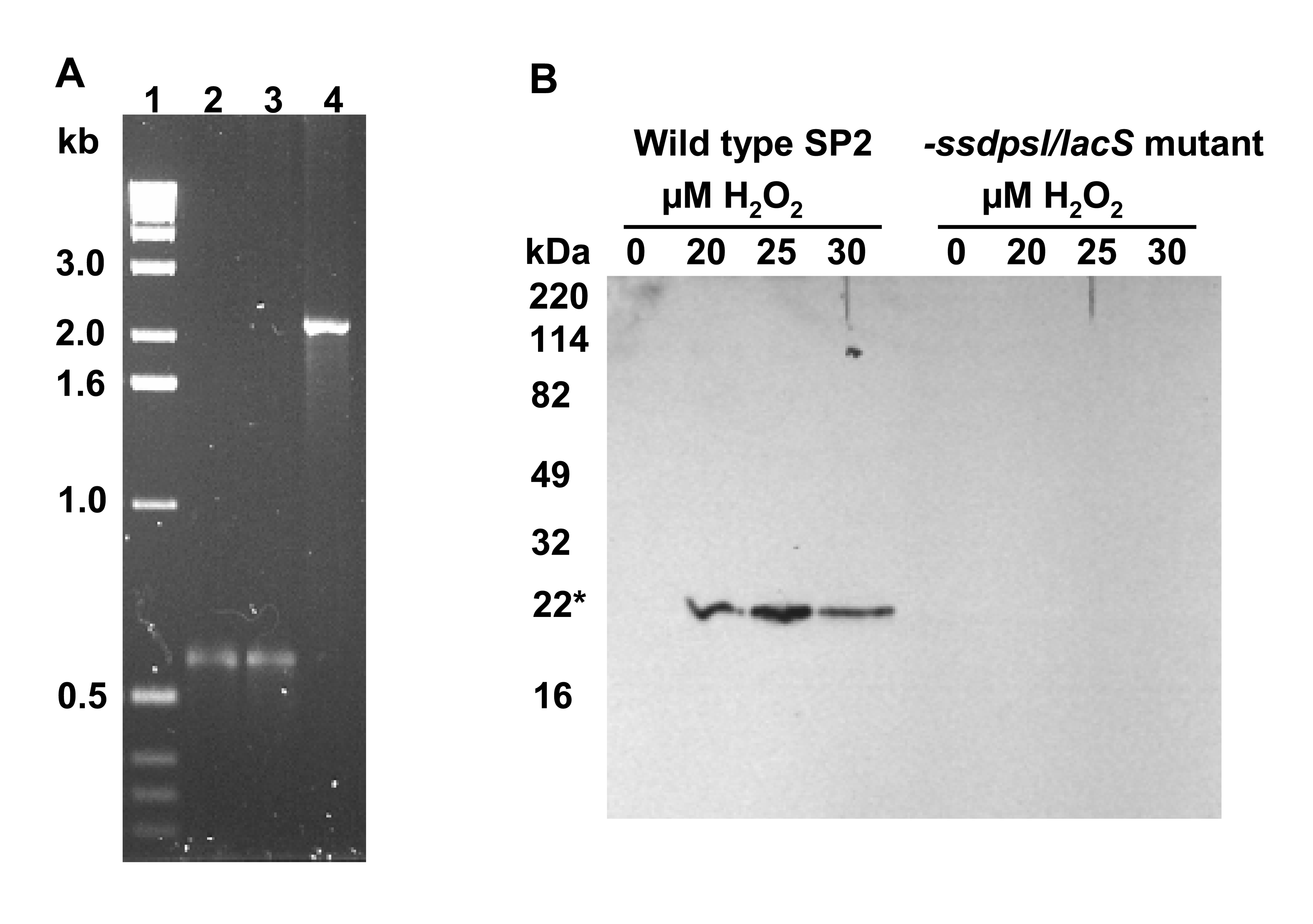

Supplement: Figure S1 — LacS disruption mutant of the ssdpsl gene in S. solfataricus. A. PCR amplification of the dpsl gene from genomic DNA isolated from, Lane 2) S. solfataricus, strain P2; Lane 3) S. solfataricus strain 98/2; Lane 4) lacS insertion into the S. solfataricus strain 98/2 dpsl gene. DNA sequencing identified a single nucleotide difference between the S. solfataricus P2 and 98/2 dpsl genes. B. Western Blot performed on wild type and dpsl mutant cells stressed with 0, 20, 25 and 30 µM H2O2. Approximately 8 µg of protein was loaded in each lane and electrophoretically separated on a 15% SDS-polyacrylamide gel. Proteins were transferred to a nitrocellulose membrane and probed with polyclonal antibodies raised against purified recombinant SsDPSL protein. Star indicates the 22kDa SsDPSL induced protein. (0.90 MB TIF) [file pone.0006964.s001.tif]
